# Draft genome sequences for *Pantoea ananatis* ATCC 35400 and *Pantoea stewartii* subspecies *indologenes* ICMP 10132

**DOI:** 10.1128/MRA.00471-23

**Published:** 2023-11-20

**Authors:** David A. Baltrus, Gi Yoon Shin, Teresa Coutinho, Brian H. Kvitko

**Affiliations:** 1 School of Plant Sciences, University of Arizona, Tucson, Arizona, USA; 2 Department of Plant Pathology, University of Georgia, Athens, Georgia, USA; 3 Department of Biochemistry, Genetics, and Microbiology, University of Pretoria, Pretoria, South Africa; 4 The Plant Center, University of Georgia, Athens, Georgia, USA; Department of Biology, Queens College, , New York, USA

**Keywords:** *Pantoea ananatis*, *Pantoea stewartii*, phage-derived bacteriocins

## Abstract

Here, we describe draft genome sequences for two bacterial isolates from the genus *Pantoea*. *Pantoea ananatis* ATCC 35400 was originally isolated from honeydew melon and was obtained from the American Type Culture Collection. *Pantoea stewartii* subspecies *indologenes* ICMP 10132 was originally isolated from sugarcane and classified as *Pantoea ananatis*, but average nucleotide identity and discriminatory PCR support species reclassification.

## ANNOUNCEMENT

**Fig 1:**
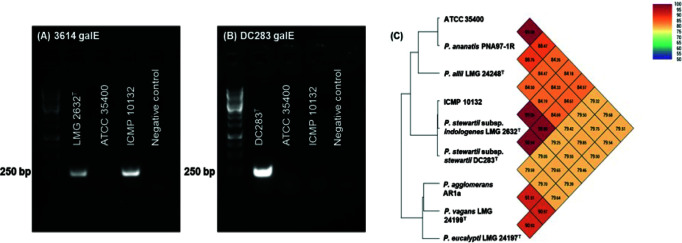
*Pantoea* strain ICMP 10132 was identified as *P. stewartii* subsp. *indologenes* using *P. stewartii* subspecies-specific PCR (3). (**A**) *P. stewartii* subsp. *indologenes*-specific 3614 *galE* PCR showed a positive band (250 bp) for the reactions containing the genomic DNA of *P. stewartii* subsp. *indologenes* type strain and ICMP 10132, whereas (**B**) *P. stewartii* subsp. *stewartii*-specific DC283 *galE* PCR yielded a positive band (250 bp) for the reaction carrying the genomic DNA of *P. stewartii* subsp. *stewartii*-type strain DC283 only. Species identification of *Pantoea* strains ATCC 35400 and ICMP 20132 using OrthoANI (4) revealed that the strains ATCC 35400 and ICMP 10132 are *P. ananatis* and *P. stewartii* subspecies *indologenes*, respectively. Over 95% average nucleotide identity was shared between the genomes of strains belonging to ATCC 35400 and *P. ananatis* PNA 97-1R, as well as the genomes of ICMP 10132 and two subspecies of *P. stewartii*.

*Pantoea* bacteria are widely distributed throughout many environments and are often found in association with or as pathogens of numerous plant species (1). We recently described a new phage-derived bacteriocin locus present within a subset of *Pantoea* isolates (2) and characterized pathways for the production of these molecules from two different strains: *Pantoea ananatis* ATCC 35400 and *Pantoea stewartii* subspecies *indologenes* ICMP 10132. Here, we present draft genome sequences for these strains. Strain ICMP 10132 is designated as *Pantoea ananatis* within the ICMP culture collection, but subspecies discriminatory PCR (3) and whole genome comparisons using default parameters in OrthoANI (4) support its classification as *Pantoea stewartii* subspecies *indologenes* (Fig. 1).

The sequenced isolates were obtained from the Culture Collection (ATCC 35400) and the International Collection of Micro-organisms from Plants (ICMP 10132) and underwent minimal passaging (<5 passages) prior to genomic DNA extraction. For genomic DNA extraction, each strain was streaked to isolation from frozen stocks onto lysogeny broth (LB) agar plates and grown at 27°C for 2 days, at which point a single colony was picked to 2 mL LB and grown overnight at 27°C and 220 rpm. Genomic DNA was extracted from overnight cultures using a Promega (Madison, WI, USA) Wizard Kit following the manufacturer’s instructions and with the inclusion of RNAse treatments. Each sequencing library described below is the product of an independent genomic DNA extraction following this protocol.

Illumina libraries were created and sequenced for both strains at SeqCenter (Pittsburgh, PA, USA) following the standard workflow for library preparation and read trimming. As described in reference (5), this workflow uses an Illumina tagmentation kit for library generation, followed by sequencing on a NextSeq 550 instrument with 150 bp paired-end reads. Trimmomatic (6) was used for adaptor trimming. This workflow generated a total of 1,788,440 paired reads and 453,777,691 bp total sequence for *P. ananatis* ATCC 35400 and 1,846,096 paired reads and 450,907,558 bp total sequence for *P. stewartii* ICMP 10132.

Oxford nanopore libraries were created and sequenced for both strains of Plasmidsaurus (Eugene, OR, USA) following the standard workflow for library preparation. Samples were prepared using the Oxford Nanopore Technologies Ligation Sequencing Kit version SQK-LSK114 and sequenced on GridION 10.4.1 flowcells (FLO-MIN114) with the “Super accuracy” basecaller in MinKNOW using ont-guppy-for-promethion version 6.5.7. This workflow generated a total of 14,855 reads (59,413,255 bp total, N_50_ of 4.288 kb) for *P. ananatis* ATCC 35400 and 39,583 reads (151,417,052 bp total, N_50_ of 3.98 kb) for *P. stewartii* ICMP 10132. An additional nanopore library was prepared for *Pantoea ananatis* ATCC 35400 using the Oxford Nanopore Technologies Ligation Sequencing Kit version SQK-LSK109 and sequenced on a MinION using 9.4 flowcells (FLO-MIN106) with basecalls in “fast” mode Guppy version 3.2.6 using a MinIT device (ont-minit-release 19.10.3). This workflow generated a total of 66,841 reads (85,739 bp total, N_50_ of 1.67 kb). Neither sample was sheared or underwent size selection prior to library preparation.

Draft genomes for both strains were assembled using Unicycler version 0.4.8 (7). For *P. ananatis* ATCC 35400, both long read files were concatenated prior to assembly. Only contigs larger than 200 bp were uploaded to GenBank, such that the draft genome assembly of *P. ananatis* ATCC 35400 consists of 13 contigs that total 4.6 Mb (N_50_ of 2.4 Mb) and that of *P. stewartii* ICMP 10132 consists of 13 contigs that total 5.2 Mb (N_50_ of 4.21 Mb). Sequences were annotated using the NCBI PGAP (8). Default parameters were used for all software.

## Data Availability

This Whole Genome Shotgun project for *P. ananatis* ATCC 35400 has been deposited in DDBJ/ENA/GenBank under accession no. JARNMU000000000. The version described in this paper is the first version, JARNMU010000000. Raw reads used to generate this assembly can be found at SRX19856715, SRX19856716, and SRX19856717. This Whole Genome Shotgun project for *P. stewartii* ICMP 10132 has been deposited in DDBJ/ENA/GenBank under accession no. JARNMT000000000. The version described in this paper is the first version, JARNMT010000000. Raw reads used to generate this assembly can be found at SRX19854976 and SRX19854977.
